# Effects of Hot Water Immersion at Different Temperatures during a Transition Period on Vertical Jump Performance

**DOI:** 10.5114/jhk/215290

**Published:** 2026-04-02

**Authors:** Caiyan Li, Anjie Wang, Chansol Hurr

**Affiliations:** 1 Integrative Exercise Physiology Laboratory, Department of Physical Education, Jeonbuk National University, Jeonju, South Korea.; 2 School of Physical Education and Health, Chengdu University of Traditional Chinese Medicine, Chengdu, China.; 3 Department of Physical Education, Anhui Polytechnic University, Wuhu, China.

**Keywords:** passive warming, muscle temperature, exercise performance, transition phase, warm-up

## Abstract

Increased muscle temperature (Tm) enhances exercise performance but can decrease during the transition period between the warm-up and the competition. Although passive warming strategies can maintain Tm, the optimal heating temperatures and their effects on performance are unclear. Seventeen healthy males participated in this study with four experimental conditions: no intervention (CON), hot water immersion at 36°C (HWI_36_), 39°C (HWI_39_), and 42°C (HWI_42_) during a 20-min transition period. Vertical jump (VJ) performance, core temperature (Tc), the heart rate (HR), and perceived thermal comfort were measured across eight VJ sets (VJ1−8) performed over 1 h, and biomechanical analyses were conducted to understand the changes in jump performance. Significant improvements in VJ performance were observed immediately after the transition period under all hot water immersion (HWI) conditions compared to CON (CON 49.2 ± 4.7 vs. HWI_36_ 50.9 ± 4.8 vs. HWI_39_ 52.2 ± 3.8 vs. HWI_42_ 53.5 ± 4.4 cm, all p < 0.05 for VJ2). HWI_39_ and HWI_42_ sustained greater VJ performance up to 50 min post-immersion than did CON and HWI_36_ (all p < 0.05 for VJ2–7). HWI_39_ and HWI_42_ increased knee range of motion and peak knee angular acceleration in the concentric phase during jumps (p < 0.05). Tc and the HR were significantly higher under the HWI_42_ condition (p < 0.05), with participants reporting greater thermal discomfort. HWI_39_ and HWI_42_ during the transition period enhanced and sustained VJ performance, with HWI_39_ being more tolerable. The enhanced VJ performance was primarily due to improved range of motion and concentric muscle contraction. These findings provide valuable insights for the optimization of passive warming strategies in competitive sports.

## Introduction

Warming up prior to competitive exercise is crucial for achieving optimal performance (Pojskic et al., 2024), largely due to temperature-related mechanisms (McGowan et al., 2015). There is a strong association between power output and muscle temperature (Tm), such that a 1°C increase in Tm can enhance subsequent exercise performance by approximately 2–5% (Racinais and Oksa, 2010). Elevation of Tm is known to provide various physiological benefits, including increased contraction speed (Lee et al., 2024), muscle fiber relaxation (De Ruiter and De Haan, 2000; Kurt et al., 2024), anaerobic metabolic capacity (Burnley et al., 2001), and nerve conduction speed (Tillin and Bishop, 2009). In many sports events, however, players are only permitted a 10–30 min rest interval between the warm-up and the start of the competition, which is called the transition period (Fairbank et al., 2021; McGowan et al., 2016). Importantly, elevated Tm following a warm-up declines rapidly and returns to near-resting values within 15–20 min (Cowper et al., 2024), such that the performance benefit from the warm-up exercise can decline during this period (Silva et al., 2025). Therefore, researchers have investigated various passive warming strategies administered during the transition period to maintain the elevated Tm gained from the warm-up. Previous studies have shown that passive warming between the active warm-up and the competition can improve short-duration explosive exercise performance in various activities, including swimming (McGowan et al., 2016; West et al., 2013), repeated VJs (Wang et al., 2023), and repeated sprint performance (Fairbank et al., 2021).

However, despite the potential benefits of passive warming, there are currently no standardized guidelines for what constitutes an optimal heat dose for passive warming. Indeed, a variety of passive heating modalities (e.g., local heating pads, HWI, heated garments) (McGowan et al., 2016; Rodrigues et al., 2020; Wang et al., 2023) with a wide range of heating temperatures (i.e., 36°C and 42°C) (Bailey et al., 2012; Pournot et al., 2011) has been shown to effectively enhance exercise performance. Importantly, temperature is a crucial factor in the effectiveness of passive warming; however, no studies have directly compared the effects of different passive heating temperatures applied during the transition period on subsequent exercise performance. Furthermore, an important yet frequently neglected issue is determining the duration of the performance benefit following passive warming. A recent investigation demonstrated that active warm-ups at high intensity could improve jump performance and maintain enhanced performance for longer duration than warm-ups at moderate intensity (Chiba et al., 2022). However, to the best of our knowledge, no study has investigated the time-course effects of passive warming on exercise performance. Rodrigues et al. (2020) examined Tm kinetics during and after lower-body HWI and found that HWI at 42°C for 2 h increased Tm by 2.8°C and it returned to the baseline level after ~116 min of recovery. However, exercise performance was not assessed following HWI; furthermore, it is not feasible to intervene in the 2-h HWI prior to the competition.

The present study aimed to fill this research gap and examine the effects of passive warming at different temperatures during a 20-min transition period on VJ performance. Additionally, it evaluated the sustained effects of passive warming at various temperatures over the course of 1 h. This aspect is particularly relevant in competition settings, where athletes often encounter variable delays between the warm-up and performance (e.g., call room procedures in track & field, staggered starts in swimming). To explore the mechanisms underlying the altered VJ performance in response to passive warming, biomechanical analyses were conducted, focusing on the knee angle and angular acceleration during the eccentric and concentric phases of the VJ. We hypothesized that passive warming at higher temperatures during the transition period would enhance performance over a longer period than passive warming at lower temperatures.

## Methods

### Participants

Seventeen healthy young males (age: 26.4 ± 3.3 yrs, body height: 177.2 ± 6.8 cm, body mass: 73.8 ± 9.6 kg, fat-free mass: 64.2 ± 9.6 kg, fat content: 18.2 ± 3.0%) who regularly participated in various exercises, including running, cycling, swimming and resistance training (5.1 ± 2.3 h per week), participated in this study, representing a recreationally active but non-competitive training status. All participants were free of cardiovascular or metabolic disease and musculoskeletal injuries, and none had prior experience with hot water immersion protocols. Participants were instructed to refrain from strenuous physical activity, alcohol consumption, and caffeine ingestion for 24 h prior to the experimental sessions.

### Design and Procedures

This study employed a randomized crossover design. Participants attended five laboratory sessions, including one familiarization session and four data collection sessions. During the familiarization session, after participants provided both verbal and written informed consent, we introduced them to the experimental procedure and equipment. Additionally, we obtained anthropometric characteristics such as body mass, body height, fat-free mass, and the fat percentage. Following the familiarization session, participants underwent four experimental sessions with four different interventions during a 20-min transition period prior to the VJ test: (1) resting in a sitting position as a control condition (CON), (2) HWI at 36°C (HWI_36_), (3) HWI at 39°C (HWI_39_), and (4) HWI at 42°C (HWI_42_). These four visits were assigned in randomized order with at least a 48-h interval between sessions. The order of the four experimental conditions was randomized using a computerized random sequence generated with the RAND function in Microsoft Excel (simple randomization), and the allocation was concealed from participants until the start of each session. For each participant, all sessions occurred at the same time of day (± 1 h). The temperature in the laboratory environment remained constant at 16°C.

**Figure 1:**
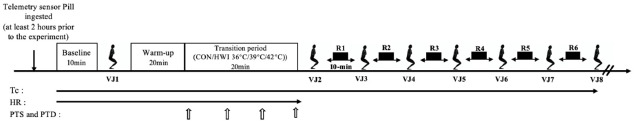
Experimental protocol schematic. HWI, Hot water immersion at 36°C, 39°C, and 42°C; VJ 1–8, vertical jump trial 1–8; R1–6, 10-min of passive recovery between each set; Tc, core body temperature; HR, heart rate; PTS, perceived thermal sensation; PTC, perceived thermal comfort

Figure 1 presents a schematic representation of the experimental procedure. Upon arrival at the laboratory, each participant rested for 10 min (baseline). During the baseline period, reflective markers were strategically positioned on key anatomical points on the participants’ lower limbs, and the baseline core body temperature (Tc) was measured. The procedure for defining Cardan angles (Davis et al., 1991) was used to determine the joint angles and reconstruct the embedded coordinate system from 0° markers at the three lower extremity joints (hip, knee, and ankle) in the standing position; markers were added to the back, hip, knee, and ankle joints for motion analysis (Wang et al., 2023). The Tc recorder (CorTemp®, HQ Inc, Palmetto, FL, United States) was wirelessly connected with the sensor pill and placed on the participant’s waist to allow for continuous recording of their core temperature. Following the manufacturer’s instructions, participants were instructed to ingest the capsules at least 2 h before the start of each intervention and were not allowed to consume any food 3 h prior to all study visits (Byrne and Lim, 2007; Domitrovich et al., 2010).

Following the 10-min baseline period, participants performed the first VJ test (VJ 1) and then completed a standardized 20-min warm-up consisting of four phases: rise, activation, mobilization, and potentiation (i.e., RAMP warm-up) (Ciesluk et al., 2024). An additional 5-min warm-up replicated the protocol of Fairbank et al. (2021) which comprised jogging and dynamic stretches (high knees, heel flicks, walking leg swings, walking lunges, and mountain climbers [2 × 30 s]). All warm-ups were supervised by the same researcher to ensure consistency. Next, a 20-min transition period was provided under four different conditions. For three HWI conditions, a bathtub (polypropylene, NT122, 138 × 62 × 52 cm) was filled with 220 L of water that was heated continuously with an electric heater to maintain the temperature at 36°C, 39°C, and 42°C. To control the hydrostatic pressure of water immersion, we selected 36°C for thermoneutral immersion as a control condition without significant thermal stress (Brunt et al., 2016). Participants sat motionless in a bathtub and were immersed up to the level of the anterior superior iliac spine. Researchers continuously monitored the water temperature using two electronic thermometer probes. Subsequently, participants performed seven sets of the VJ test (VJ 2–8) with a 10-min passive rest interval (sitting on a chair) allowed between subsequent sets. Three jumps were performed in each set with a 10-s rest interval between jumps. Tc was continuously recorded and averaged over the last 60 s of each phase, and the readings were not accessible to participants. Perceived thermal sensation was assessed on a scale ranging from 4 (neutral) to 8 (unbearably hot), whereas perceived thermal comfort was measured on a scale from 1 (comfort) to 5 (extreme discomfort) every five min during the transition phase (Rodrigues et al., 2020).

### Measures

Participants were instructed to jump to the highest point that they could reach for all jumps. To minimize the influence of arm swinging, participants crossed their arms and placed their hands on their shoulders. This approach had been previously recommended to restrict upper body involvement, thereby providing a more accurate assessment of lower-limb explosive power (Morrison et al., 2004). All participants wore the same shoes throughout the testing period, and air-cushioning shoes were not allowed. To prevent potential feedback effects, participants were not given access to jump height measurements during the test. The height of each jump was calculated as the mean value of the three jumps in each set, an approach shown to provide high reliability in vertical jump testing (Rodriguez-Rosell et al., 2017). Jump height, the knee angle, and angular acceleration during the VJ were measured using a 3D motion tracking system featuring 13 infrared cameras (Prime 17 W, OptiTrack, Natural Point, Inc., Corvallis, OR, USA) with a 1,000 Hz sampling frequency. To measure the ground reaction force produced during the VJ, force data were collected at 360 Hz using a force plate implanted in the ground (AMTI Inc., Newton, MA, USA). Synchronized data collection from the motion capture system and the force plate was performed using Motive 2.2.0 software (OptiTrack, Natural Point, Inc., Corvallis, OR, USA).

### Statistical Analysis

All data are expressed as the mean ± standard deviation (SD). Normality was assessed using the Shapiro-Wilk test, and all variables were normally distributed. Two-way repeated-measures analysis of variance (ANOVA) was used to determine the main effects of condition, time, and condition × time, followed by Tukey’s post hoc analysis. The alpha for significance was set a priori at *p* < 0.05 for all comparisons. Partial eta-squared (η2) was calculated to estimate the effect size of the two-way ANOVA (main effects and interaction), with thresholds of 0.01, 0.06, and > 0.14 representing small, medium, and large effects, respectively (Lakens, 2013). All analyses were performed using GraphPad Prism 9 (GraphPad Software, San Diego, CA, USA), which automatically applied the Greenhouse-Geisser correction when the sphericity assumption was violated.

## Results

**Figure 2:**
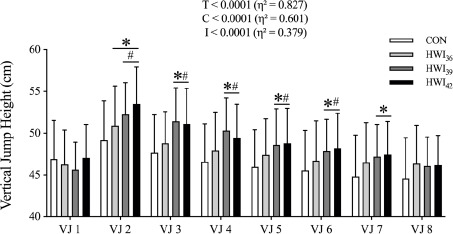
Vertical jump height. HWI, Hot water immersion at 36°C (HWI_36_), 39°C (HWI_39_), and 42°C (HWI_42_); VJ 1–8, vertical jump trial 1–8; data are presented as mean ± SD, n = 17; * p < 0.05 vs. CON, ^#^ p < 0.05 vs. HWI_36_

Figure 2 shows changes in VJ height. Significant main effects of time and condition, as well as interaction, were observed on jump height (*p* < 0.0001 for all comparisons; η^2^ = 0.827, η^2^ = 0.601 and η^2^ = 0.379). The results demonstrated an increase in VJ height for HWI_36_, HWI_39_, and HWI_42_ conditions, respectively, when compared to the CON condition following the transition period (VJ 2, CON vs. HWI_36_, *p* = 0.0065, ES = 0.37; CON vs. HWI_39_, *p* = 0.0003, ES = 0.73; CON vs. HWI_42_, *p* < 0.0001, ES = 0.97; VJ 3, CON vs. HWI_39_, *p* < 0.0001, ES = 0.88; CON vs. HWI_42_, *p* < 0.0001, ES = 0.78; VJ 4, CON vs. HWI_39_, *p* < 0.0001, ES = 0.89; CON vs. HWI_42_, *p* = 0.0002, ES = 0.66; VJ 5, CON vs. HWI_39_, *p* = 0.0013, ES = 0.59; CON vs. HWI_42_, *p* < 0.0001, ES = 0.65; VJ 6, CON vs. HWI_39_, *p* = 0.0022, ES = 0.53; CON vs. HWI_42_, *p* < 0.0001, ES = 0.59; VJ 7, CON vs. HWI_39_, *p* = 0.0252, ES = 0.54; CON vs. HWI_42_, *p* = 0.0019, ES = 0.59). Furthermore, jump height was significantly increased under HWI_39_ and HWI_42_ conditions compared to the HWI_36_ condition for VJ 2–6 (VJ 2, HWI_36_ vs. HWI_39_, *p* = 0.0093, ES = 0.31; HWI_36_ vs. HWI_42_, *p* = 0.0036, ES = 0.57; VJ 3, HWI_36_ vs. HWI_39_, *p* < 0.0001, ES = 0.68; HWI_36_ vs. HWI_42_, *p* = 0.0004, ES = 0.59; VJ 4, HWI_36_ vs. HWI_39_, *p* = 0.0001, ES = 0.56; HWI_36_ vs. HWI_42_, *p* = 0.0622, ES = 0.34; VJ 5, HWI_36_ vs. HWI_39_, *p* = 0.0259, ES = 0.27; HWI_36_ vs. HWI_42_, *p* = 0.0443, ES = 0.32; VJ 6, HWI_36_ vs. HWI_39_, *p* = 0.0340, ES = 0.27; HWI_36_ vs. HWI_42_, *p* = 0.0230, ES = 0.33). There was no significant difference between the HWI_39_ and the HWI_42_ condition during each jump (*p* > 0.05).

Table 1 shows changes in the flexion angle. During the eccentric contraction phase, significant main effects of time and condition, as well as interaction, were observed on the flexion angle (*p* < 0.0001, *p* < 0.0001 and *p* = 0.0001; η^2^ = 0.788, 0.633, and 0.236). During the concentric contraction phase, significant main effects of time and condition were observed on the extension angle (*p* < 0.0001 for all comparisons; η^2^ = 0.865, 0.553, and 0.330). During the eccentric contraction phase, significant main effects of time and condition were observed on peak angular acceleration (*p* < 0.0001 and *p* = 0.0483; η^2^ = 0.183 and η^2^ = 0.117). However, no significant differences were observed among the four conditions during the eccentric phase. During the concentric contraction phase, significant main effects of time and condition were observed for peak angular acceleration (both *p* < 0.0001; η^2^ = 0.801 and η^2^ = 0.556). Each HWI condition led to increased peak angular acceleration during the concentric phase. Additionally, there was no significant interaction in peak angular acceleration for the eccentric and concentric phases (*p* = 0.8271 and *p* = 0.5141, respectively).

**Table 1:** Range of motion and peak knee angular acceleration during the vertical jump.

			CON	HWI_36_	HWI_39_	HWI_42_
Range of motion	EccentricPhase(°)	VJ 1	71.8 ± 15.3	73.3 ± 15.2	73.9 ± 14.2	73.9 ± 14.8
		VJ 2	78.6 ± 14.6	81.2 ± 14.5*	84.5 ± 13.5*^#^	87.0 ± 14.6*^#^
		VJ 3	76.4 ± 15.1	78.8 ± 14.3*	82.2 ± 13.9*^#^	83.9 ± 13.4*^#^
		VJ 4	74.0 ± 14.6	76.5 ± 14.4*	80.7 ± 13.8*^#^	81.2 ± 13.2*^#^
		VJ 5	72.6 ± 14.8	75.6 ± 13.9*	79.5 ± 14.1*^#^	81.0 ± 13.0*^#^
		VJ 6	71.7 ± 14.8	74.6 ± 14.0*	78.5 ± 14.2*^#^	79.1 ± 13.3*^#^
		VJ 7	70.8 ± 14.6	73.4 ± 14.2*	75.0 ± 13.6*	75.6 ± 13.9*
		VJ 8	70.5 ± 14.6	72.0 ± 14.3	72.8 ± 14.2	72.9 ± 14.0
	ConcentricPhase(°)	VJ 1	98.0 ± 13.2	98.5 ± 12.8	97.8 ± 14.5	100.0 ± 15.6
		VJ 2	104.5 ± 11.9	106.5 ± 12.2*	109.7 ± 12.3*^#^	114.4 ± 13.6*^#^
		VJ 3	101.4 ± 13.3	104.7 ± 12.7*	108.0 ± 12.0*^#^	110.3 ± 12.2*^#^
		VJ 4	100.0 ± 13.1	103.3 ± 12.8*	105.9 ± 12.1*^#^	106.0 ± 11.6*^#^
		VJ 5	98.3 ± 13.1	101.5 ± 12.3*	103.8 ± 11.8*^#^	104.7 ± 11.9*^#^
		VJ 6	96.0 ± 12.0	97.8 ± 12.9*	101.9 ± 12.2*^#^	103.8 ± 11.6*^#^
		VJ 7	95.6 ± 12.9	95.6 ± 12.1	97.2 ± 11.3	97.2 ± 11.4
		VJ 8	93.0 ± 12.2	91.6 ± 9.9	94.5 ± 11.3	94.0 ± 11.3
Peak knee angular acceleration	EccentricPhase(°/s^2^)	VJ 1	30893 ± 7031	31964 ± 3667	31128 ± 4812	31996 ± 6275
		VJ 2	32290 ± 6253	33747 ± 6313	34669 ± 6721	34729 ± 7837
		VJ 3	32747 ± 5867	33791 ± 5819	34731 ± 7243	34512 ± 7859
		VJ 4	31654 ± 6623	33474 ± 6172	33841 ± 6440	34054 ± 7533
		VJ 5	32046 ± 5378	33060 ± 4830	33700 ± 6862	34186 ± 7346
		VJ 6	31262 ± 5578	33053 ± 7985	33291 ± 4990	32451 ± 6176
		VJ 7	31250 ± 5558	33141 ± 5103	33319 ± 6077	32966 ± 7297
		VJ 8	29637 ± 5456	31986 ± 3165	31546 ± 4863	31132 ± 5911
	ConcentricPhase(°/s^2^)	VJ 1	113591 ± 14752	117769 ± 14286	118469 ± 13223	119170 ± 12298
		VJ 2	130661 ± 14747	134951 ± 13399*	140185 ± 11726*^#^	139912 ± 14913*^#^
		VJ 3	127951 ± 15967	131757 ± 14292*	135400 ± 12666*^#^	134398 ± 13129*
		VJ 4	124630 ± 15976	129499 ± 13734*	132576 ± 13373*^#^	131648 ± 13228*
		VJ 5	122629 ± 16142	127282 ± 14119*	130210 ± 13453*	130012 ± 13391*
		VJ 6	119911 ± 14982	124665 ± 13046*	128061 ± 13315*	128172 ± 12699*^#^
		VJ 7	117721 ± 15711	122985 ± 14175*	126395 ± 13056*	126819 ± 12984*^#^
		VJ 8	114992 ± 15343	118813 ± 14798	123849 ± 12683	123821 ± 12716

Data are presented as mean ± SD, n = 17; * p < 0.05 vs. CON, ^#^ p < 0.05 vs. HWI_36_

Table 2 lists the force development rates. Significant main effects of time and condition, as well as interaction, were observed in the RFD (*p* < 0.0001 for all comparisons; η^2^ = 0.875, η^2^ = 0.737, and η^2^ = 0.379). There were significant enhancements under each HWI condition compared with the CON condition.

**Table 2:** Rate of force development (RFD).

		CON	HWI_36_	HWI_39_	HWI_42_
RFD(N/s)	VJ 1	1372.6 ± 247.3	1359.9 ± 219.6	1379.4 ± 229.2	1372.0 ± 231.6
	VJ 2	1501.7 ± 243.8	1582.0 ± 232.8*	1716.7 ± 249.4*^#^	1741.7 ± 243.9*^#^
	VJ 3	1410.5 ± 231.4	1497.4 ± 228.8*	1652.4 ± 228.6*^#^	1672.1 ± 239.7*^#^
	VJ 4	1387.4 ± 263.6	1477.6 ± 222.8*	1635.7 ± 213.8*^#^	1608.4 ± 214.3*^#^
	VJ 5	1357.9 ± 231.4	1410.4 ± 219.8	1568.4 ± 193.4*^#^	1521.8 ± 213.5*^#^
	VJ 6	1341.5 ± 247.5	1373.6 ± 203.8	1485.4 ± 207.1*	1473.4 ± 208.8*^#^
	VJ 7	1336.9 ± 244.5	1360.1 ± 203.8	1387.2 ± 221.0	1391.2 ± 217.7
	VJ 8	1308.0 ± 247.0	1344.7 ± 205.8	1367.1 ± 214.8*	1372.0 ± 231.6

Data are presented as mean ± SD, n = 17; * p < 0.05 vs. CON, ^#^ p < 0.05 vs. HWI_36_

**Figure 3:**
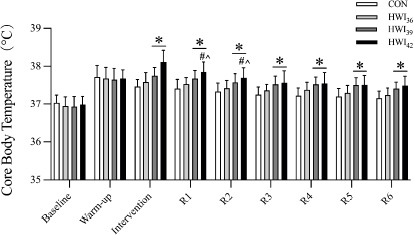
. Core body temperature. R1–6 represent each rest interval between sets. Data are presented as mean ± SD, n = 17; * p < 0.05 vs. CON. ^#^ p < 0.05 vs. HWI_36_. ^ p < 0.05 vs. HWI_39_

Figure 3 illustrates changes in Tc. Significant main effects of time and condition, as well as interaction, were observed on Tc (*p* < 0.0001, *p* = 0.0137, *p* < 0.0001; η^2^ = 0.832, η^2^ = 0.576, and η^2^ = 0.429). There was no significant difference in Tc between the baseline and the warm-up period under all conditions (all *p* > 0.05). The results showed higher Tc under the HWI_39_ compared to the CON condition and higher Tc under the HWI_42_ compared to the other three conditions at the end of immersion (CON vs. HWI_39_, *p* = 0.0278, ES = 0.56; CON vs. HWI_42_, *p* = 0.0009, ES = 0.80; HWI_36_ vs. HWI_42_, *p* = 0.0116, ES = 0.70; HWI_39_ vs. HWI_42_, *p* = 0.0006, ES = 0.58.)

Table 3 presents changes in perceived thermal sensation (PTS) and thermal comfort (PTC). Significant main effects of time and condition were observed in PTS and PTC (PTS, *p* = 0.0437 and < 0.000; η^2^ = 0.0130 and 0.849; PTC, *p* = 0.0450 and <0.0001, η^2^ = 0.264 and 0.763, respectively). However, there was no significant difference in the interaction between PTS and PTC (*p* = 0.3863 and *p* = 0.7470, respectively). The results showed higher PTS values and higher PTC values under the HWI_42_ than under the other conditions during the immersion (all *p* < 0.05).

**Table 3:** Perceived thermal sensation (PTS) and thermal comfort (PTC).

		HWI_36_	HWI_39_	HWI_42_
PTS	5 min	4.2 ± 0.4	5.3 ± 0.5^#^	6.4 ± 0.5^#^^
	10 min	4.3 ± 0.5	5.4 ± 0.5^#^	6.6 ± 0.8^#^^
	15 min	4.3 ± 0.7	5.6 ± 0.7^#^	6.5 ± 0.9^#^^
	20 min	4.8 ± 0.8	5.7 ± 0.5^#^	6.5 ± 0.8^#^^
PTC	5 min	1.0 ± 0.0	1.3 ± 0.5	2.3 ± 0.5^#^^
	10 min	1.2 ± 0.4	1.4 ± 0.5	2.3 ± 0.5^#^^
	15 min	1.3 ± 0.5	1.7 ± 0.8	2.7 ± 0.8^#^^
	20 min	1.4 ± 0.5	1.8 ± 0.6	3.0 ± 1.0^#^^

PTS (score range: 4–8) and PTC (score range: 1–5); data are presented as mean ± SD, n = 17; # p < 0.05 vs. HWI36, ^ p < 0.05 vs. HWI_39_

## Discussion

The objective of the current study was to examine the effect of hot water immersion (HWI) at different temperatures during a 20-min transition period on vertical jump (VJ) performance and to investigate the time-course effect of passive warming. The current data show that applying passive warming during this period can enhance VJ performance, with similar improvements observed following HWI_39_ and HWI_42_, which were superior to those of CON and HWI_36_ (Figure 2). Additionally, the effects of HWI_39_ and HWI_42_ on VJ performance lasted for 50 min.

Numerous studies have demonstrated that passive warming can attenuate the decrease in Tm during the transitional period, thereby enhancing subsequent exercise performance (Fairbank et al., 2021; McGowan et al., 2016; West et al., 2013). To the best of our knowledge, our study is the first to compare the effects of HWI on exercise performance at different temperatures. Without any intervention during the 20-min transition phase, the performance of VJ 2 was significantly lower in the CON group than in the other three groups submitted to HWI conditions, which suggests that the warm-up-induced performance benefit may be partially eliminated due to a decline in Tm in the CON group (Figure 2). Additionally, VJ performance was elevated following HWI_39_ and HWI_42_ compared to the CON condition throughout the 50 min following immersion, whereas HWI_36_ led to improved performance only immediately after the transition period. Thus, the current data indicate that the heating temperature of HWI during the transition period needs to be 39°C or higher in order to exhibit enhanced performance.

Our data showed that HWI application increased the range of motion during the eccentric and concentric phases of the VJ (Table 1). An increase in Tm following HWI administration increases tissue extensibility (Nakano et al., 2012) and alters the viscoelastic properties of the musculotendinous unit (Cronin et al., 2008), leading to increased range of motion in the associated joints. Increased joint range of motion facilitates a broader spectrum of force generation, which is crucial in activities that require extensive muscle engagement such as vertical jumps (Wang et al., 2023). Furthermore, peak angular acceleration at the knee during the concentric phase increased significantly in the HWI_39_ and HWI_42_ groups, whereas no significant differences were found during eccentric muscle contraction (Table 2). An increased Tm can enhance the rate of nerve impulse transmission, subsequently augmenting the speed of muscle responses and contractions (Bishop, 2003). This enhancement in nerve conduction velocity and muscular responsiveness during the concentric phase may facilitate a faster generation of force, thereby increasing angular acceleration (Wang et al., 2023). Moreover, increased muscle fluid volume caused by passive heating can change the intracellular space and the muscle fiber shape (Eng et al., 2018). These alterations potentially increase muscle force capacity (Sugi et al., 2013; Thames et al., 1974) and shorten muscle contraction time (Thames et al., 1974) by increasing the cross-bridge attachment rates (Edman and Andersson, 1968). An increase in Tm also induces intramuscular alterations (*i.e*., increased calcium influx, Ca^2+^ sensitivity, and intracellular fluid) (Blazevich and Babault, 2019) which can improve muscular force output (Racinais et al., 2017). Accordingly, our data indicated a higher RFD under HWI_39_ and HWI_42_ than under CON and HWI_36_ conditions (Table 2).

In comparison, the eccentric phase is related to VJ performance via stretch-induced gains in muscle function (Aboodarda et al., 2013). During eccentric contraction, the effect of Tm becomes more complex (Warren et al., 2002). Although an increase in Tm may boost the overall responsiveness of the muscle, force production during eccentric contractions is primarily constrained by the muscle’s structural characteristics and proficiency of neuromuscular coordination (Armstrong et al., 2022; Merrigan et al., 2021). Together, the current data indicate that the improved VJ performance following HWI application seems to be mainly attributable to an increased range of motion and enhanced concentric muscle contraction leading to increased force output rather than to mechanisms associated with eccentric muscle contraction.

Our data showed that core body temperature (Tc) increased by 0.10 ± 0.08°C under the HWI_39_ and by 0.55 ± 0.03°C under the HWI_42_ condition in response to HWI relative to the post warm-up. A recent study showed a similar result where the Tm of the vastus lateralis and rectal temperature increased to 0.90 ± 0.10°C and 0.13 ± 0.01°C, respectively, above the baseline value 15 min after commencing HWI at 42°C (Rodrigues et al., 2020). While an increased Tm may enhance muscle contractile function, increases in core temperature can impair the central drive to working muscles during voluntary contractions (Racinais and Oksa, 2010). For example, there are reports of a decrease in maximal voluntary contraction torque during moderate (*i.e*., Tc = 38.5°C) (Todd et al., 2005) and severe hyperthermia (*i.e*., Tc = 39.5°C) (Periard et al., 2014). Tc did not exceed 38.5°C under any conditions in our study (Figure 3), which presumably did not influence central drive and exercise performance. Under the HWI_42_ condition, however, Tc increased up to 38.12 ± 0.3°C immediately after HWI. Given the cool environment of our study (16°C), it is plausible that Tc would become higher than 38.5°C in a thermoneutral or a hot environment. Therefore, further investigations are warranted.

Perceived thermal sensation and comfort were measured during HWI to assess thermal tolerance at different temperatures. In the present study, participants reported thermal sensation at the end of the HWI period as close to “hot” and “very hot” under HWI_39_ and HWI_42_ conditions, respectively (Table 3). Previous studies targeting a Tc of 39.5°C during passive heating reported this experience as “very uncomfortable” (Cheung and Sleivert, 2004) and “extremely uncomfortable” (Morrison et al., 2009). In our study, participants reported their thermal comfort below “slightly uncomfortable” under the HWI_39_ condition and close to “uncomfortable” under the HWI_42_ condition, which aligns with the results of a previous investigation (Rodrigues et al., 2020). Thus, thermal sensation and comfort suggest that HWI_39_ is more tolerable than HWI_42_. Taken together, these findings indicate a potential ceiling effect, whereby physiological benefits plateau around 39°C, and further heating to 42°C may be offset by perceptual discomfort and neuromuscular limitations.

This study has several limitations. First, intramuscular temperature was not measured in the current study. Since muscle temperature is a more direct determinant of exercise performance than core temperature, simultaneous recording of both would further elucidate the effects of passive warming on physiological responses and performance. Additionally, the feasibility of HWI as a passive warm-up strategy is thought to be limited as showering or HWI in the last 10–20 min before the start of the competition is impractical (Bishop, 2003; McGowan et al., 2015). Therefore, past research has mainly focused on more practical modalities such as heated athletic garments and blizzard survival jackets (Fairbank et al., 2021; McGowan et al., 2016). Nevertheless, the current findings regarding the effects of heating temperature and the duration of effectiveness may provide valuable insights into a variety of applicable passive warming strategies. Finally, it is worth noting that only males were included in the present study. It has been suggested that women who receive the same exogenous heat stress as men have a greater increase in Tc owing to differences in physical characteristics (i.e., smaller body mass and higher fat mass) and sweat rates (Iyoho et al., 2017). Future studies are warranted to investigate the sex differences in the effects of HWI on performance.

## Conclusions

The results of this study highlight the benefits of HWI as a passive warming strategy for athletes seeking to maintain or enhance VJ performance during the transition period between the warm-up and the competition. Specifically, immersing in water at 39°C and 42°C during a 20-min transition period can enhance immediate jump performance and sustain it for up to 50 min post-immersion, with improvements linked to increased knee range of motion and peak knee angular acceleration. However, HWI at 39°C appears to be the optimal temperature for balancing performance gains and thermal comfort, as 42°C immersion, while effective, may lead to thermal discomfort and higher core temperatures. While the direct application of HWI before competition may be limited due to logistical constraints, coaches and athletes can adapt these findings to more feasible passive heating methods, such as heated garments, to maintain muscle readiness and enhance explosive performance. These findings provide a practical basis for selecting heating temperatures that maximize performance benefits while minimizing discomfort, ultimately helping athletes achieve competitive advantage through refined warm-up protocols.

## Data Availability

The datasets generated during and/or analyzed during the current study are not publicly available, but are available from the corresponding author who was an organizer of the study.
